# Acupotomy for nerve entrapment syndrome: A systematic review protocol

**DOI:** 10.1097/MD.0000000000018327

**Published:** 2019-12-16

**Authors:** Yifeng Shen, Ting Li, Tao Cai, Juan Zhong, Jing Guo, Huarui Shen

**Affiliations:** a Hospital of Chengdu University of Traditional Chinese Medicine, Chengdu; b College of Pharmacy, Southwestern Medical University, Luzhou City; c Chengdu Sport University, Chengdu; d Department of Joint Surgery, Affiliated Traditional Chinese Medicine Hospital of Southwest Medical University, Luzhou City, Sichuan Province, PR China.

**Keywords:** acupotomy, nerve entrapment syndrome, protocol, systematic review

## Abstract

**Background:**

Acupotomy has been widely used to treat nerve entrapment syndrome. But its efficiency has not been scientifically and methodically evaluated. The aim of this study is to evaluate the efficacy and safety of the acupotomy treatment in patients with nerve entrapment syndrome.

**Methods:**

Fifteen databases will be searched from inception to Dec 2019. We will include randomized controlled trials (RCTs) assessing acupotomy for nerve entrapment syndrome. All RCTs on acupotomy or related interventions will be included. Study inclusion, data extraction and quality assessment will be performed independently by 2 reviewers. Assessment of risk of bias and data synthesis will be performed using RevMan 5.3 software. Cochrane criteria for risk-of-bias will be used to assess the methodological quality of the trials.

**Results:**

This study will provide a high-quality synthesis of pain VAS and functional disability or the quality of life, the success treatment rate, the recurrent rate and the complications rate to assess the effectiveness and safety of acupotomy for nerve entrapment syndrome patients.

**Conclusion:**

This systematic review will provide evidence to judge whether acupotomy is an effective intervention for patients with nerve entrapment syndrome.

**PROSPERO registration number:**

CRD42018109086.

## Abstract 2

Supplemental Digital Content is available in the text

## 1 Introduction

Peripheral nerve disorders comprise a gamut of problems that significantly affect patient function and quality of life. These disorders include entrapment neuropathy, such as carpal tunnel syndrome. Nerve entrapment syndromes usually have typical clinical presentations and findings on physical examination. Imaging can be used to evaluate a structural cause of the entrapment, such as a mass or enlarged muscle or to show secondary findings that confirm the diagnosis, such as nerve swelling or muscle edema or atrophy.^[1]^ The common mechanisms of injury are compression, traction, ischemia and laceration.^[2]^ A peripheral nerve may become entrapped anywhere along its course, but certain anatomic locations are characteristic. Clinically, nerve entrapment is divided into three stages: in stage I patients feel rest pain and intermittent paresthesias which are worse at night; in stage II, continued nerve compression leads to paresthesias, numbness, and, occasionally, muscle weakness that does not disappear during the day, and in stage III, patients describe constant pain, muscle atrophy, and permanent sensory loss.^[3]^

Mild degree injuries associated with closed injuries are typically managed expectantly without surgical intervention.^[4]^ Typically, nonoperative therapy is recommended for at least 3 months and consists of a trial of anti-inflammatory or pain medication, splinting, avoidance of exacerbating activities or positions, physical therapy, and local steroid injections. If worsening symptoms (despite nonoperative treatment), severe symptoms, or advanced findings (ie, significant atrophy) are observed at the time of initial presentation, operative intervention should be considered. Surgery consists of decompression of the nerve, combined, at times, with other procedures to provide a better path of or bed for the nerve (eg, transposition).^[5]^

Acupotomy, also referred to as mini-scalpel needle or needle-knife, is one complementary and integrative medicine modality that modernizes acupuncture by combining conventional acupuncture needle and small-knife.^[6]^ It has been used as a tool for minimally invasive operative management for decades. The origin of the treatment is “Nine Classical Needles” from the era of Huangdi's Internal Classic (Huangdi's Internal Classic, Huang Di Nei Jing); the treatment was developed into a modernized tool, acupotomy, by Zhu Hanzhang in 1976.^[7]^ Nowadays, Acupotomy has been widely used clinically by doctors of traditional Chinese Medicine, orthopedics and pain department to treat nerve entrapment syndrome China with satisfied efficacy.^[8–11]^ Korean scholars also introduced acupotomology into clinical treatment.^[12,13]^ Acupotomy is widely used for musculoskeletal conditions, clinical evidence suggests that this treatment can relax muscular spasm and relieve compressed nerves and vessels by using the small-knife to detach taut muscle bands.^[14,15]^

However, the effectiveness of acupotomy for nerve entrapment syndrome remains controversial. This study adopts the method of evidence-based medicine to analyze and evaluate clinical RCTs in patients with nerve entrapment syndrome, in order to provide evidence for further enhancing the clinical curative effect on patients with nerve entrapment syndrome. The study will assess the effectiveness and safety of the acupotomy treatment in nerve entrapment syndrome patients.

## 2 Methods

### 2.1 Inclusion criteria for study selection

#### 2.1.1 Types of studies

All the RCTs of acupotomy for the management of nerve entrapment syndrome patients will be included without publication status restriction or writing language.

#### 2.1.2 Types of patients

Inclusion criteria for study populations will be all patients with nerve entrapment syndrome. No restrictions will be applied in terms of gender, race, and education status.

#### 2.1.3 Types of interventions and controls

*Experimental interventions:* The treatment group will be treated with acupotomy (there is no limit on the needle materials, treatment methods, and course of treatment).

*Control interventions:* Because there is no false acupotomy reported in the literature and acupotomy commonly used in the acupuncture-moxibustion department. The control group will adopt the internationally recognized therapy such as block therapy or no treatment, acupuncture will also be included. Acupotomy with another active therapy vs the same therapy alone will also be investigated. Studies comparing 2 different types of acupotomy or surgical procedures will be expelled.

#### 2.1.4 Types of outcome measures

*Primary outcomes:* Improvement in pain, as measured by the visual analogue scale (VAS) or other validated pain scoring system if VAS is not used.

*Secondary outcomes:* The secondary outcomes are reduction in other scales or questionnaires evaluating pain or functional disability or the quality of life; The success treatment rate (after treatment the participants with a reduction of scales =50% comparing to baseline), the recurrent rate and the complications rate.

### 2.2 Search methods for the identification of studies

#### 2.2.1 Data sources

Electronic databases will be searched from their inception and will include Cochrane Central Register of Controlled Trials, PubMed, MEDLINE, EMBASE, and 4 Chinese databases (China National Knowledge Infrastructure, Chinese Biomedical Literature Database, VIP Database and Wanfang Database), 6 Korean databases (Korean Studies Information, DBPIA, Korean Institute of Science and Technology Information, KERIS, KoreaMed, Korean National Assembly Library) and the Japanese database (CiNii Articles). We will also conduct non-electronic searches of conference proceedings, our own article files. The search strategy that will be applied in the MEDLINE database is presented in Appendix A. Similar search strategies will be used in the other databases. We will also search the reference lists of review articles and identify RCTs for any possible titles matching the inclusion criteria.

#### 2.2.2 Searching other resources

The authors will scan the reference lists and retrieve additional studies. In addition, authors will search the WHO International Clinical Trials Registry Platform (ICTRP) (http://apps.who.int/trialsearch/) and Google Scholar (http://scholar.google.co.kr/). Dissertations of degrees will be included. The ClinicalTrials.govregistry (http://clinicaltrials.gov/) will be searched for any unpublished trials.

### 2.3 Data extraction, quality, and validation

#### 2.3.1 Study inclusion

**Figure 1:**
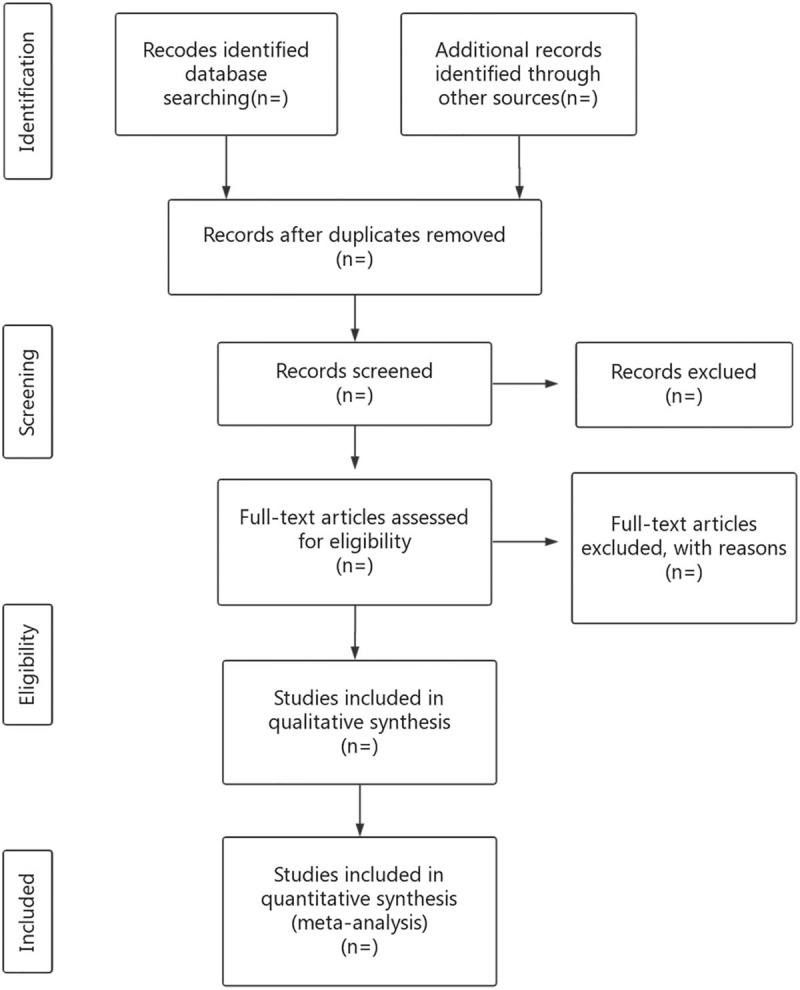
Flow diagram of study selection process.

Researchers will import the literature retrieved to the EndnoteX7 and eliminate the duplicate data. The noticeably below-standard articles will be deleted by reading the title and abstract. After that, the researchers will read the full text, discuss in the group, and contact the author for research details to determine the final inclusion of the literature (Fig. 1). The final list of articles will be converted into Microsoft Excel format. Two researchers will independently conduct the literature search and literature screening. Finally, another study member will resolve the inconsistencies and check the final literature that will be included.

#### 2.3.2 Data extraction and management

Data from the selected articles will be extracted and filled by 2 reviewers independently in the data extraction form. Any disagreement will be solved by consensus or an arbiter. We will extract information such as reference ID, author, time of publication, characteristics of participants, blinding, interventions, follow-up, outcome indicators, research results, adverse events, and other detail information. We will be in contact with the authors of trials for further information when necessary.

### 2.4 Risk of bias assessment

The risk of bias will be evaluated by 2 reviewers based on the Cochrane collaboration's tool from 7 dimensions: random sequence generation, allocation concealment, the blinding method for patients, researchers and outcomes assessors, incomplete result data, and selective reports. The terms ”Low“, ”Unclear“, and ”High“ will be referred to low, uncertain, and high risks of bias, respectively. In most cases, disagreements will be settled by discussion between the 2 reviewers. If disagreement remained after discussion, a third reviewer will be consulted before taking the final decision on the disagreements.

### 2.5 Quantitative data synthesis and statistical methods

#### 2.5.1 Quantitative data synthesis

In our review, meta-analysis will be performed using software RevMan 5.3. For dichotomous data, we will present results as risk ratio (RR) with 95% confidence intervals (CIs). For continuous data, mean difference (MD) will be included in the meta-analysis. If outcome variables are measured on different scales, standard mean differences (SMD) analysis with 95% CIs will be included in the meta-analysis.

#### 2.5.2 Assessment of heterogeneity

The *χ*^2^ test will be used to assess statistical heterogeneity. The *I*^2^ test will be used to quantify the inconsistencies between the included studies. If the *I*^2^ value is less than 50%, the study will not be considered heterogeneous while a value greater than 50% would indicate significant heterogeneity.

#### 2.5.3 Assessment of reporting biases

When more than 10 trials are included in the study, the funnel plot will be used to detect potential reported biases. When the image is not clear, the STATA 11.0 software will be quantified using the EGRATER test.

#### 2.5.4 Subgroup analysis and investigation of heterogeneity

If there is a significant heterogeneity in the included trials, we will conduct subgroup analysis based on the type of disease, differences in treatment frequencies and follow-up durations will also be included.

#### 2.5.5 Sensitivity analysis

If possible, a sensitivity analysis will be performed to verify the robustness of the review conclusions. The impact of methodological quality, sample size, and missing data will be assessed. In addition, the analysis will be repeated after the exclusion of low methodological quality studies.

#### 2.5.6 Grading the quality of evidence

We will apply the Grading of Recommendations Assessment, Development and Evaluation (GRADE) method to evaluate the level of confidence in regards to outcomes. Two independent reviewers will conduct the assessment. In most cases, disagreements were resolved by discussion between the 2 reviewers. If disagreement remained after discussion, a third reviewer will be consulted before taking the final decision on the disagreements.

## 3 Discussion

Acupotomy for nerve entrapment syndrome is a miniature surgery, with higher acceptability and less pain. It is crucial to make sure whether acupotomy is a good option for the patients. Studies have shown that acupotomy can effectively reduce the symptoms of nerve entrapment syndrome, but its efficacy has not been evaluated scientifically and systematically. The aim of this study is to evaluate the efficacy and safety of the acupotomy treatment in patients with nerve entrapment syndrome, we hope this review will provide more evidence. There are some limitations in this review. Different types of acupotomy treatment and different disease may run the risk of heterogeneity. In addition, the measurements and tools of outcomes of included studies may be different.

## Author contributions

HS is the guarantor of the article. The manuscript was drafted by YS and TC. TL and JZ developed the search strategy. TC and TL will independently screen the potential studies and extract data. JZ and JG will assess the risk of bias and finish data synthesis. HS will arbitrate any disagreement and ensure that no errors occur during the review. All review authors critically reviewed, revised, and approved the subsequent and final version of the protocol.

## Supplementary Material

Supplemental Digital Content
