# Supplementary material for: Acupotomy for nerve entrapment syndrome: A systematic review protocol
Source: Medicine (Baltimore). 2019 Dec 16;98(50):e18327. doi: 10.1097/MD.0000000000018327 (PMC6922462; doi:10.1097/MD.0000000000018327)
Supplement: Supplemental Digital Content [file medi-98-e18327-s001.doc]

**Appendix 1. Search strategy used in PubMed database**

1. “nerve compression syndromes"[Title/Abstract] OR “nerve entrapment syndrome"[Title/Abstract] OR “entrapment neuropathy”[Title/Abstract] OR “superior clunial nerve”[Title/Abstract] OR “nervus cutaneus femoris lateralis”[Title/Abstract] OR “lateral femoral cutaneous nerve”[Title/Abstract] OR “Occipital nerve”[Title/Abstract] OR “ suprascapular nerve”[Title/Abstract] OR “carpal tunnel syndrome”[Title/Abstract] OR “piriformis”[Title/Abstract]

2."acupotomy"[Title/Abstract] OR "small needle-knife"[Title/Abstract] OR "needle knife"[Title/Abstract]

3. "randomized controlled trial"[Title/Abstract] OR "controlled clinical"[Title/Abstract]

4.#1 AND #2 AND #3
